# A bibliometric analysis of studies related to the nuclear factor kappa B signaling pathway in knee osteoarthritis between 2004 and 2023

**DOI:** 10.3389/fmed.2025.1572161

**Published:** 2025-06-25

**Authors:** Yafeng Mo, Jiahao Chu, Tiewu Chen, Wenbing Liu, Jing Tang

**Affiliations:** ^1^Department of Orthopedics, Zhejiang Rehabilitation Medical Center, Hangzhou, China; ^2^Department of Orthopedics, The Third Affiliated Hospital of Zhejiang Chinese Medical University, Hangzhou, China

**Keywords:** knee, osteoarthritis, NF-κB, signaling pathway, bibliometric analysis

## Abstract

**Objective:**

This study aimed to identify the principal research areas and trends in the nuclear factor kappa B (NF-κB) signaling pathway in knee osteoarthritis (KOA).

**Methods:**

The Web of Science core collection (WoSCC) database was searched for studies related to the NF-κB signaling pathway in KOA published between 2004 and 2023. The complete records of the literature and all citations were exported from the WoSCC database to plain text file and tab-delimited file, respectively. The exported data were then analyzed. Analysis was conducted using CiteSpace (version 1.6.20) and VOSviewer (version 6.1. R6), OriginPro 2021 (version 9.8.0.200), SCImago Graphica (version 1.0.44), and the bibliometric website (http://bibliometric.com/).

**Results:**

A total of 752 studies were included in this analysis, and the results demonstrated an overall increasing trend in the number of published papers and citation frequency over the period 2004−2023. China leads in terms of the number of publications, with 387 publications, followed by the United States (118), and Japan (45). The institutions with the highest number of publications were China Medical University (28) and The Second Affiliated Hospital of Wenzhou Medical University (Yuying Children’s Hospital) (24). The most frequently occurring keywords were “articular cartilage,” “inflammation,” “activation,” and “expression.” Furthermore, recent keywords with high research intensity included “histopathology,” “mesenchymal stem cells,” “subchondral bone,” and “hip.” Toll-like receptor 4 (TLR4), monosodium iodoacetate (MIA), and high-performance anion-exchange chromatography with pulsed amperometric detection (HPAE-PAD) were popular research topics.

**Conclusion:**

The bibliometric analysis revealed that studies of NF-κB signaling pathways in KOA have predominantly focused on the TLR4/NF-κB signaling pathway and exosomes. NF-κB plays a core regulatory role in KOA, with molecular evidence supporting its involvement in inflammation, cartilage degradation, and pain signaling. However, current research is limited by the lack of *in vivo* imaging techniques to visualize NF-κB activity in real-time. Future research should prioritize the development of such imaging modalities and integrate multi-omics approaches, including single-cell and spatial transcriptomics, to analyze pathway heterogeneity and identify novel therapeutic targets. This integrative approach will facilitate a deeper understanding of the pathogenesis of KOA and enable the development of more effective treatment strategies.

## 1 Introduction

Knee osteoarthritis (KOA) is one of the most prevalent conditions in orthopedics. Lesions frequently affect articular cartilage and periarticular tissues, resulting in a gradual onset of pain and loss of knee function. In severe cases, disability may ensue, significantly affecting the quality of life of patients with KOA (1). The prevalence of KOA significantly correlates with advancing age. In the context of an aging global population, the prevalence of KOA is rapidly increasing in middle-aged and older individuals. A review of the literature reveals that KOA has a prevalence rate of approximately 22.9% among individuals aged 40 and above (2). This condition is becoming a significant global health concern that demands attention, as it places a considerable burden on healthcare systems and the families of affected individuals. KOA is not yet curable, implying a long-term medical commitment. Furthermore, late-stage use of knee replacement is more invasive, which has prompted many researchers to shift the focus of their research to the early prevention and treatment of KOA (3, 4). The precise mechanism underlying KOA development remains unclear. However, it is evident that inflammation plays a significant role in its onset. Inflammatory factors, including Interleukin-6 (IL-6), Interleukin- 1beta (IL-1β) and tumor necrosis factor-alpha (TNF-α), are instrumental in early diagnosis and treatment of KOA (5–7). The classical nuclear factor kappa B (NF-κB) pathway regulates several inflammatory factors, including IL-6, IL-1 beta and TNF- alpha (8) Inhibition of the NF-κB pathway can effectively attenuate the progression of KOA. Research into the NF-κB pathway has played an important role in the prevention and treatment of KOA, making the NF-κB pathway a key area of research in the prevention and treatment of KOA (9–11).

NF-κB plays a key role in KOA by regulating the expression of inflammatory factors (such as TNF-α and IL-17) and matrix metalloproteinases (MMPs) (12). Its crosstalk with other pathways (such as TLR4 and MAPK) adds to the complexity and significance of its role in inflammatory responses. For example, the TLR4 signaling pathway can promote the expression of inflammatory factors by activating NF-κB. NF-κB inhibitors (such as KIC-0101) have shown therapeutic potential in clinical trials, reducing the expression of inflammatory factors and alleviating KOA symptoms by inhibiting NF-κB activation (13). Future research should focus on developing more specific inhibitors to reduce side effects and enhance efficacy.

Although NF-κB, TLR4, and miRNA all play important roles in KOA, NF-κB research focuses on the regulation of inflammatory signaling pathways, TLR4 research on the recognition and transmission of inflammatory signals, and miRNA research on the regulation of gene expression (14). Activation of the NF-κB pathway leads to the overexpression of pro-inflammatory cytokines and MMPs, making it a promising target for drug development (15). IKK inhibitors and proteasome inhibitors exert anti-inflammatory effects by inhibiting NF-κB activation (16). Gene therapy, which targets key components of the NF-κB pathway, such as CRISPR/Cas9-mediated knockout of NF-κB activity to reduce inflammatory factor expression, offers new strategies for KOA treatment (17).

The activation status of NF-κB can serve as a biomarker for the diagnosis of KOA and the evaluation of treatment efficacy. By detecting the levels of inflammatory factors in blood or synovial fluid, or analyzing the expression changes of downstream target genes of NF-κB, early diagnosis and personalized treatment of KOA can be achieved. In summary, NF-κB holds great potential and clinical value in the treatment of KOA (18). Targeting the NF-κB pathway could lead to the development of new drugs and gene therapies, offering new strategies for KOA treatment.

Bibliometric analysis is a method of visualizing knowledge graphs that can be employed by researchers to identify key areas of focus, as well as the most recent theories, experimental studies, and applications in this field. Current bibliometric studies on the pathogenesis of knee osteoarthritis have provided a comprehensive picture of the potential mechanisms of knee osteoarthritis pathogenesis and the major signaling pathways involved (19). However, bibliometric analyses of NF-κB, an important signaling pathway in KOA, have yet to be published. This study addresses this gap by retrieving and analyzing all articles and reviews pertaining to the NF-κB signaling pathway in knee osteoarthritis over the past two decades. The objective of this study was to elucidate the role of the NF-κB signaling pathway in the pathogenesis of KOA and to provide an overview of cutting-edge theories and popular research directions related to the NF-κB signaling pathway in KOA. This study provides support for further research, experimental studies, and applications. Although osteoarthritis of the knee remains an incurable disease, deeper research into its mechanisms will facilitate the development of a complete cure in the near future. Therefore, this study is a crucial step in this process.

## 2 Materials and methods

**FIGURE 1:**
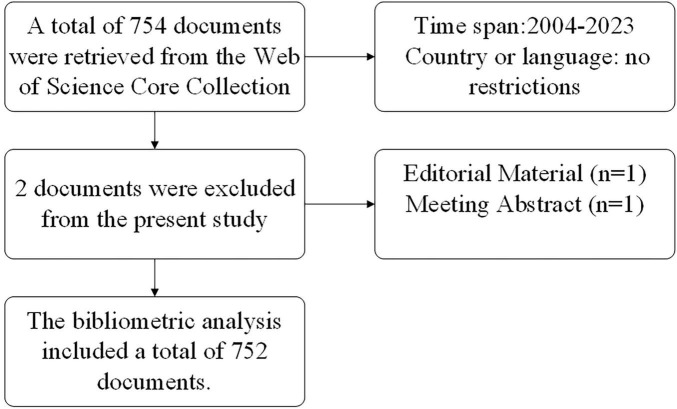
Article screening flowchart.

The Web of Science Core Collection (WoSCC) database, the most frequently selected and authoritative database for bibliometrics, was used to source literature for this study. The search was concluded on April 18, 2024, with publication dates ranging from January 1, 2004, to December 31, 2023. The search topics of this study were knee osteoarthritis and the NF-κB signaling pathways associated with it. The literature was not restricted by language or region and 754 documents were retrieved. The complete search process and the resulting search results are listed in Table 1. The full record of search results and all citations were exported from the browser in plain text files and tab-delimited files, respectively, facilitating their subsequent importation into other software for analysis. To ensure dependability of the analytical procedures, this study incorporated two categories of literature: Articles and reviews. Subsequently, the text was imported into Citespace software (version 6.3. R1) to screen and remove duplicates, resulting in 752 publications included in the study. Figure 1 provides a visual representation of the screening process.

**TABLE 1:** The topic search query.

Set	Searching keywords	Outcome
#1	TS = (NF-kappa B OR NF-κB OR Nuclear factor kappa B OR Nuclear transcription factor-κB), Indexes = WoSCC	213,060
#2	TS = (Osteoarthritis, knee OR Osteoarthritis of the knee OR Knee Osteoarthritis OR Osteoarthritis of knee OR Knee OA OR Knee, Osteoarthritis Of OR KOA), Indexes = WoSCC	65,073
#3	#1AND#2	790
#4	Time span = 2014.01.01–2023.12.31 (publications are not restricted by country or language)	754

### 2.1 Data analysis

The annual number of publications and annual citation frequency information pertaining to NF-κB signaling pathway research in KOA are presented as a composite graph of bar charts and line graphs, generated through the search result visualization function of the WoSCC database. Furthermore, the top 10 journals in terms of number of publications were identified from the WoSCC database. CiteSpace was the primary tool used to visualize the data in this study. It is professional software for bibliometric analysis developed by Dr. Chaomei Chen, which enables the presentation of information extracted from the literature in the form of a graph (20). In this study, CiteSpace software was employed for the extraction of author-related information (author, institution, country), citation-related information (reference, cited author, cited journal), and keywords from the publications. In this software, the nodes were filtered based on the g-index with a value of *K* = 25. The pruning modes employed were the pathfinder, sliced networks, and merged networks. The CiteSpace software settings were retained as they were originally configured, with the exception of the aforementioned alterations. To present the findings in a visual format, we employed CiteSpace software to generate a visual map of cited authors, cited literature, a dual-map overlay of citing and cited journals, a timeline map of the cited literature, and a Keyword Burst plot. This study also employed the JAVA-based bibliometric software, VOSviewer (version 6.1. R6) to visualize the co-occurrence relationships between authors, institutions, journals, and keywords. To clearly illustrate the evolution of the number of publications over time for countries that are significant contributors to the NF-κB signaling pathway in KOA, we employed OriginPro 2021 (version 9.8.0.200) software to map the area stacking of annual publications for the top five countries in terms of the number of publications. VOSviewer software and SCImago Graphica (version 1.0.44) software were integrated to create a map of the global distribution of countries and regions where the NF-κB signaling pathway in KOA was studied. To demonstrate the collaborative relationships among countries and regions, we employed a bibliometric platform^1^ to visualize international collaboration networks.

## 3 Results

### 3.1 Annual publications

**FIGURE 2:**
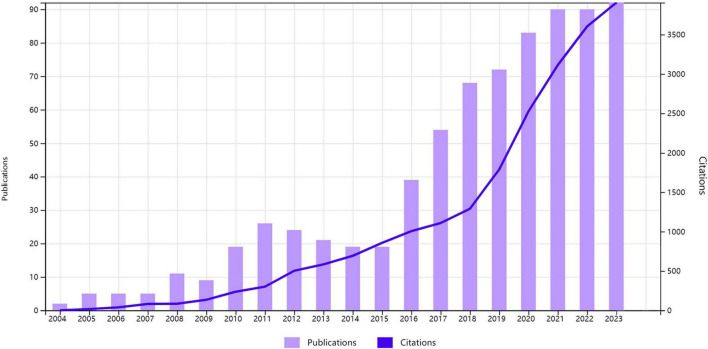
Graph of annual number of publications and frequency of citations between 2004 and 2023.

The annual publication (Figure 2) provides evidence of the evolution of research activity within the field, with 752 articles and reviews published over a 20-year period from 2004 to 2023. The number of publications showed a consistent upward trend from 2004 to 2014. The number of publications began to increase exponentially in 2015, and by 2021, it had risen nearly five-fold, reaching a peak of 20 years (19 publications to 90 publications). The number of publications remains at a peak level until 2023. Furthermore, the frequency of citations in the field demonstrated a discernible upward trajectory, suggesting that research in the field is being acknowledged by an increasing number of researchers. It seems reasonable to posit that research activities in this field will continue at a high level in the coming years.

### 3.2 Institutions and countries

CiteSpace software was employed to extract institutional information from the literature, and the results demonstrated that 402 institutions contributed to the study of the NF-κB signaling pathway in KOA. The top 10 academic institutions in terms of the number of published works are listed in Table 2. The China Medical University was the most prolific contributor to this research, with a total of 28 studies. This was followed by The Second Affiliated Hospital of Wenzhou Medical University (Yuying Children’s Hospital), which contributed 24 studies, and the Central Hospital of Wuhan, Tongji Medical College, Huazhong University of Science and Technology (19 studies). The significance of an object within a given field is gauged by the frequency of citations and Betweenness Centrality (BC). Objects with a BC value greater than 0.1 or with a high citation frequency, are of significant consequence within the field. CiteSpace software was employed to calculate the BC values of the institutions, resulting in the top 10 institutions in terms of BC values (Table 3). The combination of institutional publication volume and BC value demonstrated that the Central Hospital of Wuhan, Tongji Medical College, Huazhong University of Science and Technology was the most influential institution (BC = 0.04) in the field of NF-κB research in KOA.

**TABLE 2:** The Top 10 Institutions ranked by frequency.

Rank	Number of publications	Institution
1	28	China Medical University
2	24	The Second Affiliated Hospital and Yuying Children’s Hospital of Wenzhou Medical University
3	19	The Central Hospital of Wuhan, Tongji Medical College, Huazhong University of Science and Technology
4	18	Sir Run Run Shaw Hospital, Zhejiang University School of Medicine
5	13	Shanghai Ninth People’s Hospital, Shanghai Jiao Tong University School of Medicine
6	12	Frontier Institute of Science and Technology, Xi’an Jiaotong University
7	11	Beijing University of Chinese Medicine
8	10	Guangzhou University of Chinese Medicine
9	9	Nanjing Medical University
10	8	Renmin Hospital of Wuhan University

**TABLE 3:** The top 10 institutions ranked by centrality.

Rank	Centrality of publications	Institution
1	0.04	The Central Hospital of Wuhan, Tongji Medical College, Huazhong University of Science and Technology
2	0.04	Sir Run Run Shaw Hospital, Zhejiang University School of Medicine
3	0.04	Renmin Hospital of Wuhan University
4	0.04	The Seventh Affiliated Hospital, Sun Yat-sen University
5	0.04	The First Affiliated Hospital of Chongqing Medical University
6	0.04	University of Science and Technology of China
7	0.03	Beijing University of Chinese Medicine
8	0.03	Shenzhen Institute of Advanced Technology, Chinese Academy of Sciences
9	0.03	The Second Xiangya Hospital of Central-South University
10	0.03	Zhejiang Chinese Medical University

**FIGURE 3:**
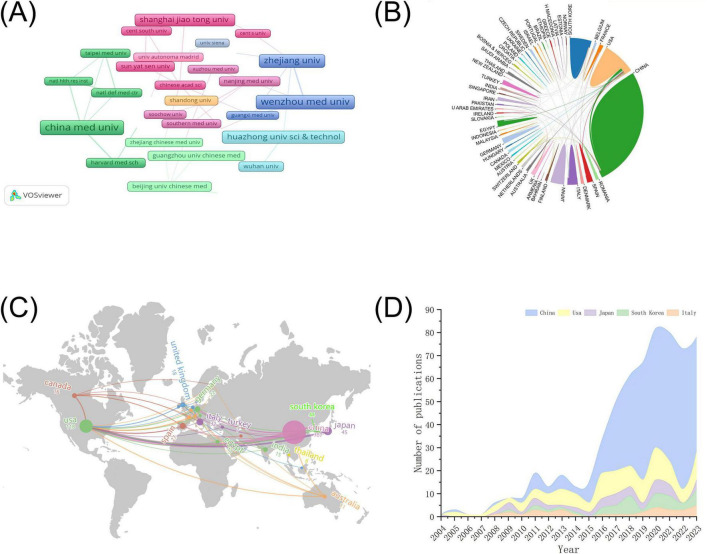
Countries (Regions) and institutions for NF-κB signaling pathway research in KOA over the past two decades infographic. **(A)** Institutional collaboration map for NF-κB pathway research at KOA. The size of the rectangles shows the number of papers issued by the institution. The connecting lines and the width of the connecting lines indicate the presence and intensity of cooperation between them. **(B)** National collaboration map for NF-κB Pathway Research at KOA. The blocks represent countries, with the size of the block indicating the number of publications. The lines between the plots show the countries’ collaborative relationships. **(C)** KOA’s world map of NF-κB pathway research publications. On the map, the size of the circle represents the number of published works. The connecting lines between different countries indicate instances of bilateral or multilateral cooperation. **(D)** Area stacked map of annual publication of the five countries with the highest number of publications in the field of NF-κB pathway research at KOA. The size of the area is indicative of the number of publications issued.

To visualize the collaboration between institutions, we also plotted the co-occurrence of institutions using VOSviewer software (Figure 3A). We set the minimum number of publications of institutions to five and finally showed the cooperation between 42 institutions. As illustrated in the figure, the majority of highly productive research organizations are based in China, with the majority of their partnerships also occurred in China.

The Country Collaboration Map (Figure 3B) provides a visual representation of the contribution share and collaboration between individual countries in the NF-κB research area in the KOA. This image illustrates the extensive scope of international collaborations among diverse nations. Figure 3C illustrates the geographical distribution of countries contributing to this field of research using the form of a map and indicating the number of publications by countries that have made a significant impact in this area. The results demonstrated that the NF-κB signaling pathway in KOA is predominantly concentrated in Asia, North America, and Europe. A list of the 10 countries with the highest number of publications is provided in Table 4, which shows that Asia exhibits a pronounced predominance in the number of publications, with over half of the literature originating from China (387), followed by the United States (118) and Japan (45). Figure 3D is an area-stacking map of the five countries with the highest number of publications, which illustrates the variation in publication numbers from year to year in each country. The number of publications in China has increased year-on-year, assuming a leading position in terms of publication output since 2016.

**TABLE 4:** The top 10 countries ranked by frequency.

Rank	Publications	Country/region
1	387	China
2	118	United States
3	45	Japan
4	44	South Korea
5	32	Italy
6	27	Spain
7	22	Germany
8	18	The United Kingdom
9	16	Canada
10	15	India

### 3.3 Journals and cited journals

Data on the number of publications in the journals were retrieved from the search results of the WoSCC database. The 10 journals with the highest number of publications are listed in Table 5. The most impactful journal in the table is *Osteoarthritis and Cartilage*, an English language publication with an impact factor (IF) of 7.2.

**TABLE 5:** Top 10 journals in terms of publications.

Rank	Journal	Publications	Impact Factor (2023)	Country
1	Osteoarthritis and Cartilage	31	7.2	England
2	International Journal of Molecular Sciences	28	4.9	Switzerland
3	International Immunopharmacology	21	4.8	Netherlands
4	Arthritis Research and Therapy	17	4.4	England
5	Scientific Reports	16	3.8	England
6	Biomedicine and Pharmacotherapy	15	6.9	France
7	Molecular Medicine Reports	15	3.4	Greece
8	Frontiers in Pharmacology	14	4.4	Switzerland
9	PLoS One	12	2.9	United States
10	Arthritis and Rheumatism	11	None	United States

The top three journals in terms of article output were *Osteoarthritis and Cartilage* (31 articles), *International Journal of Molecular Sciences* (28 articles), and *International Immunopharmacology* (21 articles). Notably, these three journals had an IF exceeding 4, reflecting their prominence in the field. The cited journal information was extracted using CiteSpace software, and the 10 most frequently cited journals are presented in Table 6. The top three most frequently cited journals, in order, are *Osteoarthritis and Cartilage* (671), *Arthritis & Rheumatism (ARTHRITIS RHEUM-US)* (498), and *Arthritis Research & Therapy* (442). *Nature Reviews Rheumatology* had the highest impact factor among the top 10 most cited journals, with an IF of 29.4.

**TABLE 6:** Top 10 most frequently cited journals.

Rank	Cited journal	Frequency	Impact factor (2023)	Country
1	Osteoarthritis and Cartilage	671	7.2	England
2	Arthritis and Rheumatism (Arthritis Rheum-US)	498	None	United States
3	Arthritis Research and Therapy	442	4.4	England
4	Annals of the Rheumatic Diseases	442	20.3	England
5	Arthritis and Rheumatism (Arthritis Rheum)	330	None	United States
6	Nature Reviews Rheumatology	319	29.4	United States
7	Journal of Biological Chemistry	314	4.0	United States
8	Journal of Rheumatology	289	3.6	Canada
9	PLoS One	278	2.9	United States
10	International Journal of Molecular Sciences	250	4.9	Switzerland

**FIGURE 4:**
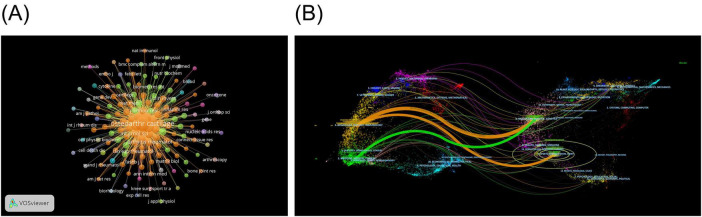
Journals and cited journals infographic for NF-κB signaling pathway research in KOA over the last two decades. **(A)** Map of KOA’s cited journals in the field of NF-κB pathway research. The nodes represent the cited journals, with the size of the node indicating the frequency of citations to the journal. The connecting lines between nodes indicate the existence of a co-occurrence relationship between the cited journals **(B)** Dual-map overlay of KOA’s journals in the field of NF-κB pathway research. The image is divided into two sections, with the left side representing the field of study to which the citing literature belongs and the right side representing the field of study to which the cited literature belongs. The bands between the two sections connects the journals in which the citation and cited relationship occurs.

The VOSviewer software was used to map the co-occurrence of the cited journals (Figure 4A). The co-citation relationships between 150 eligible cited journals were illustrated by setting the minimum citation frequency to 50. One of the journals, *Osteoarthritis and Cartilage*, exhibits a high degree of co-occurrence with other cited journals and is the most popular journal in the field of NF-κB signaling pathway research at KOA. To investigate the relationship between citing and cited areas of literature related to the NF-κB pathway in knee osteoarthritis, a dual-map overlay of citing and cited journals was conducted using Citespace. The resulting map is shown in Figure 4B. As illustrated in the graph, journals in the field of molecular/biology/immunology primarily reference publications from journals within the domains of molecular/biology/genetics and sports/rehabilitation/sport. Journals within the medicine/medical/clinical field tend to cite those within the molecular/biology/genetics field as the primary source of references.

### 3.4 Authors and cited authors

**FIGURE 5:**
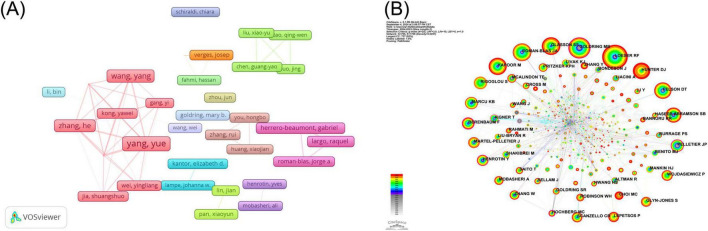
Authors and cited authors infographics for NF-κB signaling pathway studies in KOA over the last two decades. **(A)** KOA’s author collaboration mapping for the NF-κB pathway research area. The rectangles represent the authors, with the size of the rectangle indicating the number of articles published by each author. The connecting lines between the rectangles illustrate the existence of a collaborative relationship between the authors. **(B)** KOA’s cited author collaboration mapping for the NF-κB pathway research area. The nodes represent the cited authors, with the size of the nodes indicating the frequency of citation. The connecting lines between the nodes indicate the existence of a co-citation relationship between the cited authors. The authors cited with BC values exceeding 0.1 exert a greater influence, and the outermost level of the corresponding node is marked by a purple circle.

The author information of all included studies was extracted using CiteSpace software, and the 10 most prolific authors are included in Table 7. Lunhao Bai and Yue Yang, both of Chinese nationality, were the most prolific authors in this field, publishing 12 and 10 papers, respectively. To demonstrate the collaborative relationship between authors engaged in research pertaining to the NF-κB signaling pathway in KOA, we employed VOSviewer software to construct a visual map of the authors (Figure 5A). The minimum number of publications was set to four, and the collaborative relationships of the 43 authors were represented in the visualization. This analysis reveals that almost all the major contributors to the publications are part of established collaborative teams, which suggests that such teamwork is an effective strategy for enhancing the yield of research outcomes. The CiteSpace software was also employed to extract information pertaining to the cited authors from the publications, and the BC values of the cited authors were also calculated. The top five most-cited authors and top five authors with the highest BC are shown in Tables 8, 9, respectively. Richard F Loeser was the most cited (146), followed by Mary B Goldring (123) and J A Roman-Blas (106). Homas Aigner had the highest BC (0.30) and was the most influential cited author, followed by J P Pelletier (0.23) and C Bassleer (0.18). To gain insight into the relationships between co-cited authors, we employed the CiteSpace software for visualization purposes. The graph produced by the cited authors comprises 795 nodes and 1,796 connections (Figure 5B). As illustrated in the figure, there is a considerable breadth of interconnections between the cited authors.

**TABLE 7:** Top 10 authors in terms of publications.

Rank	Author	Publications
1	Lunhao Bai	12
2	Yue Yang	10
3	He Zhang	6
4	Yang Wang	6
5	Elizabeth D. Kantor	4
6	Yi Gang	4
7	Shuangshuo Jia	4
8	Jing Luo	4
9	Rui Zhang	4
10	Xiaoning Zhang	4

**TABLE 8:** Top five cited authors in terms of publications.

Rank	Frequency	Cited author
1	146	Richard F. Loeser
2	123	Mary B. Goldring
3	106	Jorge A. Roman-Blas
4	106	Sonya S. Glasson
5	103	Mohit Kapoor

**TABLE 9:** Top five cited authors in terms of BC.

Rank	Centrality	Cited author
1	0.30	Thomas Aigner
2	0.23	Jean-Pierre Pelletier
3	0.18	Corinne Bassleer
4	0.17	Xavier Ayral
5	0.15	Yves Henrotin

### 3.5 Cited references

**FIGURE 6:**
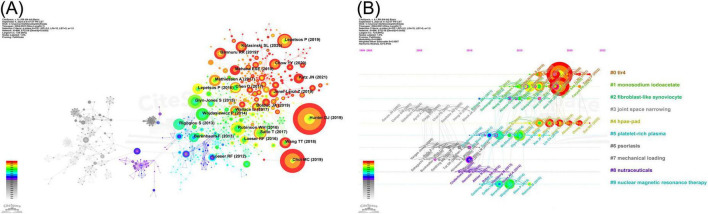
Infographic of cited literatures on NF-κB signaling pathway research in KOA over the last two decades. **(A)** Co-occurrence graph of cited literature in the field of NF-κB pathway research in KOA. The nodes represent cited literatures, with the size of the node indicating the frequency. The connecting lines between nodes represent the co-occurrence relationship between the cited literatures. **(B)** Timeline map of cited literature in the field of NF-κB pathway research in KOA. The nodes represent the cited documents, with the size of each node indicating the frequency of citations. The connecting lines between the nodes illustrate the co-citation relationship between the literature. The nodes with high impact (BC > 0.1) are marked by purple circles.

CiteSpace software was employed for the extraction of cited literature information from publications and calculation of BC values for the nodes. Cited literature was ranked according to the frequency of citations and BC values. Table 10 presents the five most frequently cited documents, whereas Table 11 displays the top five cited documents in terms of BC value. *Osteoarthritis*, published in 2019, was the most frequently cited publication (61 citations), followed by *NF-*κ*B Signaling Pathways in Osteoarthritic Cartilage Destruction* (46 citations), and *Redox and NF-*κ*B Signaling in Osteoarthritis* (29 citations). The article “*NF-kappaB Signaling: Multiple Angles to Target OA*” (BC = 0.35) is identified as the most influential, with “*Role of proinflammatory cytokines in the pathophysiology of osteoarthritis*” (BC = 0.34) ranking as a close second. The Cite Spacesoftware was used to construct a map of the cited literature. The resulting map, presented in Figure 6A, comprises 896 nodes and 2,218 links. As illustrated in the figure, the majority of the highly cited literature was published in the last decade, suggesting that research on the NF-κB signaling pathway in KOA is currently at a pivotal point in its evolution. To identify shifts in research focus over time, we employed a keyword clustering approach to group the cited literature into distinct themes. We then constructed a timeline graph (Figure 6B) to illustrate the evolutionary trajectory of these themes, showing the top 10 topics. The timeline for each theme displays a single top-cited study per year. The figure illustrates that Toll-like receptor 4 (TLR4), monosodium iodoacetate (MIA), and high-performance anion-exchange chromatography with pulsed amperometric detection (HPAE-PAD) have remained high in recent years.

**TABLE 10:** Top five Cited references ranked by frequency.

Rank	Frequency	Reference	Author
1	61	Osteoarthritis (65)	Hunter et al. (65)
2	46	NF-κB signaling pathways in osteoarthritic cartilage destruction (23)	Choi et al. (23)
3	28	Redox and NF-κB signaling in osteoarthritis (66)	Lepetsos et al. (66)
4	27	Low-grade inflammation as a key mediator of the pathogenesis of osteoarthritis (22)	Robinson et al. (22)
5	27	Osteoarthritis (4)	Glyn-Jones et al. (4)

**TABLE 11:** The top five cited references ranked by centrality.

Rank	Centrality	Reference	Author
1	0.35	NF-kappaB signaling: Multiple angles to target OA (25)	Marcu et al. (25)
2	0.34	Role of proinflammatory cytokines in the pathophysiology of osteoarthritis (67)	Kapoor et al. (67)
3	0.24	A current review of molecular mechanisms regarding osteoarthritis and pain (21)	Lee et al. (21)
4	0.19	American College of Rheumatology 2012 recommendations for the use of non-pharmacologic and pharmacologic therapies in osteoarthritis of the hand, hip, and knee (68)	Hochberg et al. (68)
5	0.19	The adverse effects of diabetes on osteoarthritis: Update on clinical evidence and molecular mechanisms (69)	King et al. (69)

### 3.6 Keywords

The frequency of the occurrence of keywords in the publications was extracted using CiteSpace software, and subsequently, the BC values of the keywords were calculated. Table 12 presents the 10 keywords with the highest frequency of occurrence. Table 13 lists the ten keywords with the highest BC values. Following the removal of search terms, the most prevalent keyword was identified as “expression,” with a total of 182 occurrences. This is followed by “inflammation” (136 times), “cartilage” (132 times), “activation” (109 times), “articular cartilage” (102 times) and “chondrocyte” (93 times). The most central keywords based on BC values were “cartilage degradation” (0.2), “tumor necrosis factor alpha” (0.15), “c reactive protein” (0.15), “collagen-induced arthritis” (0.14), “double blind” (0.13), “apoptosis” (0.13), “chondroitin sulfate” (0.12) and “*in vivo*” (0.12).

**TABLE 12:** The top 10 keywords ranked by frequency.

Rank	Frequency	Keywords
1	329	Knee osteoarthritis
2	327	NF-κB
3	182	Expression
4	136	Inflammation
5	132	Cartilage
6	109	Activation
7	102	Articular cartilage;
8	99	Knee
9	95	Osteoarthriti
10	93	Chondrocyte

**TABLE 13:** The top 10 keywords ranked by frequency and centrality.

Rank	Centrality	Keywords
1	0.20	Cartilage degradation
2	0.15	Tumor necrosis factor alpha
3	0.15	c reactive protein
4	0.14	Collagen induced arthriti
5	0.13	Chondrocyte
6	0.13	Double blind
7	0.13	Apoptosis
8	0.12	Chondroitin sulfate
9	0.12	*In vivo*
10	0.11	Disease

**FIGURE 7:**
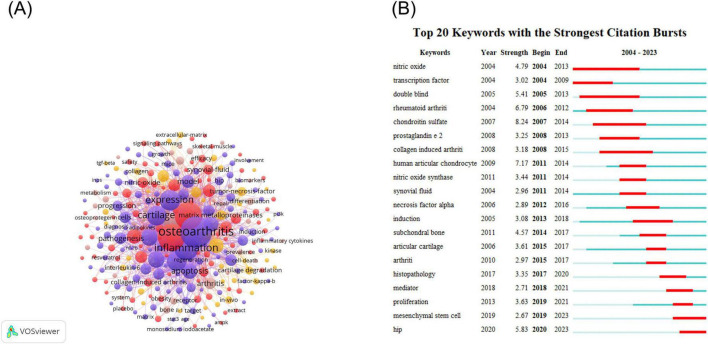
Keywords infographic for NF-κB signaling pathway research in KOA over the last two decades. **(A)** Keyword co-occurrence diagram for the NF-κB pathway research area of KOA. In the diagram, each node represents a keyword. The relative size of the nodes indicates the frequency of occurrence of the corresponding keyword. The connecting line between the nodes shows the co-occurrence relationship of the keywords. **(B)** Keyword burst ranking top 20 keywords. The color of the bar on the timeline indicates the level of research conducted on the keyword at that point in time. Red indicates that the keyword has been researched extensively, blue indicates that it has been researched to a lesser extent, and light blue indicates that it has been researched the least.

The VOSviewer software was employed to visualize the keywords of these publications, with the minimum frequency of keyword occurrence set at 5. Finally, 273 keywords were identified (Figure 7A). The analysis of keyword bursts has provided insights into the most popular research directions in recent times. To this end, we exported the top 20 keywords for keyword bursts and sorted them according to the time of burst (Figure 7B) using Citespace software. The results revealed that hip, mediator, histopathology, proliferation, and mesenchymal stem cells (MSCs) have remained highly popular from 2019 to 2023.

## 4 Discussion

The etiology of KOA remains unclear, and the condition is generally considered a common degenerative knee disorder. A review of the literature revealed several confirmed risk factors, including genetics, obesity, aging and joint malalignment (21). The primary pathological manifestation of KOA is cartilage degeneration with pain being the most prevalent clinical manifestation. Nevertheless, no substantial advancement has been achieved in the investigation of the fundamental mechanisms underlying cartilage degeneration and generation of joint pain. Consequently, the majority of current treatments are either short-term or unable to achieve the desired outcomes. Following years of research, there has been a notable shift in our understanding of KOA pathogenesis. The prevailing view that emerged from this research field is that KOA is not simply a normal degenerative change caused by joint wear and tear. Instead, it is now recognized as a multifactorial disease involving complex interplay between pro- and anti-inflammatory cytokines, chemokines, growth factors, and adipokines. Subsequent research corroborates the assertion that inflammatory processes are pivotal in this complex pathogenesis (1, 22). Unbalanced remodeling of the articular cartilage driven by inflammatory mediators, including IL-1beta, IL-6, and TNF-α, is a significant contributing factor to the development and progression of knee osteoarthritis. Notably, the production of these inflammatory mediators is regulated by the NF-κB signaling pathway (8). The NF-κB signaling pathway is activated by two distinct pathways: Canonical and non-canonical. The canonical pathway is dependent on the activation of p65, c-Rel and p50 subunits by the Inhibitor of kappa B kinase (IKK) complex (composed of IKKα, β and γ). This pathway is characterized as rapid and reversible (23) In contrast, the non-canonical pathway is distinguished by slow and persistent activation of the p52 and RelB subunits by IKKα (24). Activation of the NF-κB signaling pathway has been demonstrated to promote the expression of catabolic genes, pro-inflammatory and destructive mediators, and up-regulate transcription factors that regulate inflammatory and catabolic mediators. This results in persistent inflammation, cartilage destruction, and pain in the knee joint (25). Additionally, it also plays a pivotal role in modulating chondrocyte differentiation and cellular senescence within the knee joint (9, 10). It is therefore imperative to undertake a bibliometric analysis of the NF-κB signaling pathway, as it may prove pivotal in elucidating the intricate etiology of osteoarthritis of the knee and represents a significant step toward the potential curability of KOA.

In total, 752 papers were included in the bibliometric analysis. The popularity of a research field can be gauged to a certain extent by the number of articles published in that field. The overall trend of research related to the NF-κB signaling pathway in KOA has demonstrated a notable increase from 2004 to 2023. The number of articles published during the 2021–2023 period has remained at a consistently high level, indicating that research activity in this field has continued to be high in recent years and is likely to persist for an extended period. With regard to the number of publications, China is the leading country in this regard and represents the principal body of research in the field of NF-κB signaling pathways in KOA. As illustrated in the country and region visualization maps (Figure 3C), the regions engaged in research pertaining to the NF-κB signaling pathway in KOA are primarily concentrated in China and the United States, with extensive international collaboration. It is possible that the extensive international collaboration has been the reason for the consistently high level of research in recent years. The map of institutional cooperation (Figure 3A) demonstrates that stable cooperation has emerged between highly productive institutions within the same countries. However, this finding also reveals that international cooperation among institutions requires reinforcement. China Medical University was the most prolific contributor to publications, with the greatest number of publications from any institution. It has considerable experience in KOA research with a particular focus on exercise and mechanical stress. The most contributing author, Lunhao Bai, was employed at this institution. The classification of the source journals from which the articles originated indicated that the majority of articles were drawn from specialized journals in the fields of molecular, immunology, biomedicine, and arthritis. These journals provide researchers in related fields with professional guidance, and facilitate the development of new research ideas. The term “highly cited journals” is used to describe journals that are highly recognized within their respective fields or internationally. In this study, almost all the highly cited journals were from the fields of rheumatology and arthritis. Furthermore, most of these journals were from the United States, which suggests that the United States exerts significant scholarly influence in this field. The results of keyword co-occurrence and calculated node BC values indicated that the research themes of the NF-κB signaling pathway were primarily focused on expression, activation, inflammation, cartilage, and chondrocytes. The focus of these studies is cartilage degradation, TNF-α, C-reactive protein, apoptosis, and chondroitin sulfate (CS). The research methods employed were primarily *in vivo* and double blind. The keyword burst results indicated that the most recent research activity has focused on the relationship between the hip and knee as well as mediator, histopathology, proliferation, MSCs, and other related content. The timeline graph of the cited literature alloweds us to observe the evolution of the research topics over time. It is noteworthy that “TLR4” was only identified in 2015, yet it has been the subject of considerable interest in the field of knee osteoarthritis since that time. Furthermore, MIA and HPAE-PAD are research topics that were explored prior to the TLR4 pathway. However, the ongoing interest in these topics until the conclusion of this study indicates that MIA and HPAE-PAD remain prominent techniques and modeling drugs employed in the investigation of the NF-κB pathway in KOA.

### 4.1 NF-κB signaling pathway in KOA

NF-κB is a transcription factor that exists as a homo- or heterodimer comprising of five subunits: p65, p50, p52, c-Rel, and RelB. Among these, p65 is a major functional subunit of NF-κB (26). The NF-κB signaling system is even more intricate, comprising 15 distinct cell type- and stimulus-specific dimer combinations that exert pivotal regulatory functions in pathophysiological processes such as inflammation, immunity, cell proliferation, and differentiation (27). As a chronic inflammatory disease, the etiology of KOA is inextricably linked to the NF-κB signaling pathway. For instance, adenoviral vector-mediated NF-κBp65-specific small interfering RNA has been demonstrated to impede NF-κB activation and NF-κB p65 expression in the joint fluid of animal models. After processing, the secretion of inflammatory factors (including IL-1beta and TNF-alpha) and cartilage degradation were markedly diminished in the joint fluid of the animal model (28). Furthermore, inhibition of the upstream regulator of NF-κB activation, IKK, or the downstream target of NF-κB and cofactor E74-like factor 3 (ELF3) has also been shown to effectively reduce the degradation of articular cartilage in animal models effectively (29, 30). These results indicate that the NF-κB signaling pathway may play a pivotal role in cartilage catabolism during the progression of KOA. Imbalances in chondrocyte production and destruction have been identified as significant contributors to KOA development. The study of chondrocyte apoptosis, an important component of destructive factors, is of equal importance. Wang et al. (31) investigated the role of the NF-κB signaling pathway in the mechanism of chondrocyte apoptosis and observed that the NF-κB signaling pathway exerts two diametrically opposed roles, anti-apoptotic and pro-inflammatory, on the disease progression of KOA. This study demonstrated that the inhibition of NF-κB with caffeic acid phenethyl ester resulted in a greater number of chondrocytes undergoing apoptosis in response to the pro-apoptotic factor TNF-alpha. This phenomenon occurs concurrently with a notable reduction in the activation of pro-matrix metalloproteinase (MMP) 9, which plays an important role in inflammatory processes. Consequently, when selecting NF-κB as a potential therapeutic target for KOA, it is essential to consider the balance between its antiapoptotic and pro-inflammatory effects. Among the articles included in this study, a high-profile review article stated that the NF-κB signaling pathway promotes the activation of transcription factors, such as hypoxia-inducible factor 2 alpha, ELF3, and runt-related transcription factor 2, which in turn exacerbates cartilage breakdown of the knee joint by increasing the expression of a disintegrin and metalloproteinase with thrombospondin motifs-5 and MMP 13 proteases (32).

In clinical studies, the detection of NF-κB activity can serve as a significant indicator for assessing the inflammatory status of knee osteoarthritis (KOA). Research has shown a substantial correlation between NF-κB activity and patient pain scores. Specifically, by measuring the NF-κB DNA binding activity in synovial fluid, the degree of NF-κB pathway activation can be evaluated (23). The severity of patient pain is assessed using the Visual Analog Scale (VAS) or the Western Ontario and McMaster Universities Osteoarthritis Index (WOMAC) (33). Clinical research has found that higher NF-κB activity in synovial fluid correlates with higher patient pain scores, indicating a positive correlation between NF-κB activity and pain intensity (23, 34).

Moreover, the NF-κB signaling pathway plays a crucial role in the pathogenesis of KOA. The activation of NF-κB leads to an increase in the production of pro-inflammatory cytokines such as IL-1β and TNF-α, which are key factors in the pathological process of KOA (35). In synovial fluid, the concentration of these cytokines is closely related to the degree of inflammation, further supporting the link between NF-κB activity and the inflammatory status of KOA (36).

These research findings suggest that the detection of NF-κB activity can not only serve as an indicator for assessing the inflammatory status of KOA but also as a potential marker for evaluating treatment efficacy. By inhibiting the NF-κB pathway, the production of inflammatory cytokines can be reduced, thereby alleviating pain and improving joint function (37). Therefore, inhibitors of the NF-κB pathway may become potential therapeutic targets for the treatment of KOA (37, 38).

In recent years, in-depth studies on the NF-κB signaling pathway in KOA have led to a greater understanding of its specific pathogenesis, and the accompanying therapeutic methods have also been gradually refined. It is expected that KOA will become a curable disease in the near future.

### 4.2 TLR4/NF-κB signaling pathway and miRNA in KOA

TLRs are pattern recognition receptors capable of recognizing structures designated as pathogen-associated molecular patterns and damage-associated molecular patterns (DAMPs), which in turn trigger an immune response. TLR4, a member of the TLR family, has been demonstrated to contribute to the development of inflammation and chondrolysis in articular cartilage (39). In addition, evidence indicates that primary afferent neurons contribute to pain maintenance through the expression of TLR4. In chronic pain associated with KOA, the activation of primary sensory neurons is a crucial factor. Consequently, TLR4 may represent a promising therapeutic target for the management of chronic pain in knee (40, 41). Damage to KOA tissues results in the release of several DAMPs, including fibronectin fragments, small degradation fragments of hyaluronic acid, and high mobility group protein B1 (HMGB1) (42). The recognition of DAMPs by TLR4 results in the activation of NF-κB, which in turn leads to the assembly and activation of IL-1beta, subsequently releasing it into the joint space (43–45). And IL-1beta has been proven to accelerate cartilage degeneration and destruction. This is achieved by inhibiting the synthesis of the extracellular matrix, promoting chondrocyte apoptosis, and stimulating the expression of MMP family proteins. The net effects of these processes include development of KOA and joint pain. Zhang et al. (46) demonstrated that inhibition of TLR4/NF-κB by quercetin resulted in the down-regulation of IL-1beta and TNF-alpha expression, thereby exhibiting significant therapeutic effects in a rat model of osteoarthritis. Myeloid differentiation primary response 88 (MyD88) is a soluble bridging protein found in the cytoplasm of cells. It serves as a crucial connector molecule for the transmission of immune signals from TLR4 to NF-κB (9). A study on the relationship between obesity and arthritis revealed that saturated fatty acids activate TLR4, which initiates the recruitment of MyD88. MyD88 complexes interleukin 1 receptor-associated kinases 1 and 4, which in turn activates NF-κB (47). It can be reasonably proposed that the TLR4/MyD88/NF-κB signaling pathway may represent a pivotal signaling pathway in the pathogenesis of knee osteoarthritis. This hypothesis was initially validated through histopathological and metabolomic analyses using a rat model of papain-induced KOA. These findings demonstrate that non-weight-bearing exercise therapy effectively attenuated papain-induced joint inflammation and cartilage damage by inhibiting the TLR4/MyD88/NF-κB signaling pathway (48).

MicroRNAs (miRNAs) are short non-coding RNAs, 19–25 nucleotides in length, and their involvement in the progression of osteoarthritis (KOA) is an area of increasing research interest. These small molecules regulate gene expression at the post-transcriptional level by binding to target mRNAs, leading to their degradation or translational repression. In KOA, miRNAs are implicated in modulating inflammation, cartilage degradation, and joint pain, making them potential therapeutic targets.

For instance, Among various miRNAs, miR-382-3p has been shown to suppress the IL-1β-induced inflammatory response in KOA. IL-1β is a key pro-inflammatory cytokine that drives the pathogenesis of KOA by activating multiple signaling pathways and promoting the production of inflammatory mediators. Research has demonstrated that miR-382-3p exerts its anti-inflammatory effects by targeting connexin 43 (Cx43), thereby inhibiting the TLR4/MyD88/NF-κB signaling pathway (49). Cx43, a gap junction protein, plays a crucial role in intercellular communication and its dysregulation is associated with inflammatory processes in KOA. By reducing the overexpression of Cx43, miR-382-3p effectively blocks the TLR4 signaling cascade, leading to the inhibition of NF-κB activation and subsequent reduction in the production of inflammatory cytokines (50). This suggests that miR-382-3p holds promise as a therapeutic target for alleviating inflammation in KOA.

MiR-27a is one of the most extensively studied miRNAs in KOA. It has been consistently found to be significantly downregulated in the cartilage of KOA patients. This downregulation is closely linked to the pathophysiological features of KOA. Studies have shown that upregulating miR-27a expression can inhibit the TLR4/NF-κB signaling pathway, thereby reducing the production of inflammatory cytokines. Moreover, miR-27a promotes the synthesis of pro-cartilage repair substances, such as aggrecan and type II collagen. Aggrecan is a major component of the cartilage extracellular matrix, providing elasticity and compressive resistance to cartilage. Type II collagen is the principal structural protein of the cartilage matrix, essential for maintaining cartilage integrity and function. By enhancing the expression of these key proteins, miR-27a facilitates cartilage repair and improves joint function (49, 50). Thus, miR-27a not only plays a vital role in mitigating inflammatory responses in KOA but also promotes cartilage repair, offering a new therapeutic avenue for the disease.

MiR-515-5p has also garnered attention for its potential therapeutic role in KOA. Recent studies have revealed that miR-515-5p regulates the TLR4/MyD88/NF-κB axis via an N6-methyladenosine (m6A)-dependent mechanism (50). M6A is a prevalent RNA modification that influences various aspects of gene expression, including RNA stability, splicing, and translation. In the context of KOA, m6A modification may affect the stability and function of miR-515-5p, thereby modulating the activity of the TLR4 signaling pathway. Research has shown that upregulation of miR-515-5p can inhibit the TLR4/MyD88/NF-κB signaling pathway, resulting in the alleviation of KOA symptoms. This finding not only highlights the therapeutic potential of miR-515-5p in KOA but also underscores the importance of m6A modification in regulating miRNA function (51). Further investigation into the interplay between m6A modification and miR-515-5p could pave the way for the development of novel therapeutic strategies based on miR-515-5p, offering more treatment options for KOA patients.

HMGB1 is a nuclear protein secreted by various immune cells. The extracellular form binds to TLR4 and activates the inflammatory response. Researchers have been able to alleviate the symptoms of KOA by inhibiting the HMGB1/TLR4/NF-κB signaling pathways with the HMGB1 inhibitor glycyrrhizin, which provides a new avenue for the treatment of KOA (52). Long non-coding RNA plasmacytoma variant translocation 1 (PVT1) plays a pivotal role in intercellular communication and has been implicated in the progression of osteoarthritis (53). Meng et al. recently proposed a novel hypothesis that PVT1 in arthritis may be mediated by exosomal pathways (54). In a subsequent study, researchers constructed an *in vitro* cell injury model using lipopolysaccharide stimulation of normal human chondrocytes (C28/I2). This study demonstrated that exosomes released from injured C28/I2 cells increased PVT1 expression, whereas PVT1 depletion reversed the decreased activity, inflammatory response, apoptosis, and collagen degradation in C28/I2 cells. These findings confirm this hypothesis. Furthermore, this study revealed a negative correlation between miR-93-5p levels and PVT1 expression in osteoarthritis patients’ serum. It was subsequently demonstrated that exosome-mediated PVT1 targets miR-93-5p, modulates the activity of the HMGB1/TLR4/NF-κB pathway, and influences the progression of lipopolysaccharide-induced osteoarthritis. In a study conducted by Qiu (55), it was demonstrated that the remission of osteoarthritis resulting from IL-1beta may be attributed to the action of miR-129-5p carried by human synovial MSCs exosomes. These results indicated that the HMGB1/TLR4/NF-κB signaling pathway is the pathway through which this process is achieved.

**FIGURE 8:**
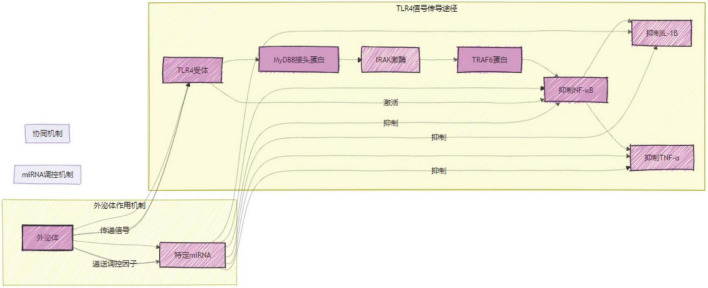
Mechanism Chart of the Synergistic Mechanism of TLR4/NF-κB, Exosomes, and miRNA.

The aforementioned studies illustrate the considerable potential of research pertaining to miRNAs, exosomes, and the TLR4/NF-κB signaling pathway in the therapeutic management of KOA (Figure 8). Therefore, further comprehensive studies are warranted.

### 4.3 CS, MIA, MSCs, and HPAE-PAD in KOA research

CS is a symptomatic slow-acting drug used to treat KOA. It is frequently used in basic and clinical experimental studies because of its efficacy in improving joint function, relieving joint pain, reducing joint space narrowing, and reducing joint swelling and effusion (43). The mechanism by which CS provides relief from KOA pain and swelling can be attributed to its anti-inflammatory effect (56). CS reduced the phosphorylation of extracellular signal-regulated kinase 1/2 and p38 mitogen-activated protein kinase in chondrocytes. This results in the inhibition of the nuclear translocation of NF-κB induced by IL-1beta, thereby exerting anti-inflammatory effects (57). The findings of this study also indicate that CS alleviates synovitis symptoms by inhibiting the nuclear translocation of NF-κB in synovial cells and macrophages. This suggests that the mechanism of action of CS in the cartilage and synovial tissues may be consistent to some extent. However, further studies are required to elucidate the precise mechanisms of action. A correlation has been established between the severity of joint pain and synovial thickening, with CS exhibiting an excellent potential to inhibit synovial inflammatory thickening, which may represent one of the underlying mechanisms involved in the reduction of joint pain (58). MIA is an inhibitor of glyceraldehyde-3-phosphate dehydrogenase activity that induces cartilage degradation, an initial inflammatory response, and pain. Consequently, it is frequently used as a modeling agent for osteoarthritis (59). In a rat model of osteoarthritis, MIA promoted the formation of reactive oxygen species and lowered the mitochondrial membrane potential (ΔΨm) in primary rat chondrocytes (60). This results in the release of cytochrome c into the cytoplasm and subsequent activation of caspase-3, which ultimately leads to apoptosis of chondrocytes in a dose-dependent manner. Notably, a transient increase in NF-κB activity at the early time points after MIA injection was demonstrated by NF-κB luminescence imaging in one study. This study provides further evidence of a positive correlation between NF-κB activity and KOA pain sensitivity (61). This allows the use of serum cytokine NF-κB luminescence imaging as a non-invasive biomarker of pain sensitivity. The above-mentioned study also revealed that NF-κB luminescence imaging can be employed for expeditious identification of targeted NF-κB antagonists. Consequently, NF-κB luminescence imaging may play a prominent role in the treatment of KOA and other NF-κB-driven pathologies. Stem cells play a pivotal role in tissue repair and regeneration strategies, demonstrating the capacity to repair cartilage damage through local promotion of stem cell expression or stem cell delivery (62). Additionally, their derived exosomes have been shown to inhibit inflammation in osteoarthritis through the HMGB1/TLR4/NF-κB pathway, a promising avenue for therapeutic intervention in KOA (55). Bone marrow MSC-derived exosomes have been demonstrated to promote chondrocyte proliferation and cartilage matrix synthesis by enhancing the expression of chondrogenic proteins and mRNA (examples include collagen type II and sex-determining region Y-box 9). This process restores the integrity and function of the articular cartilage, thereby preventing or reversing KOA development. Consequently, the study of exosomes derived from MSCs may represent a breakthrough in the reversal of KOA (63). Further in-depth studies are required to confirm this hypothesis. The quality control of food supplements and nutraceuticals used in the treatment of KOA has consistently been a subject of significant interest and research. This is due to the fact that some individuals prefer food supplements and nutraceuticals as a means of non-pharmacological treatment over pharmaceuticals. However, the food supplements and nutraceuticals are not subject to the same rigorous regulation as pharmaceutical-grade products prior to commercialization. This lack of regulations may raise concerns regarding the efficacy and safety of these products. HPAE-PAD technology represents a significant advancement in analytical chemistry, combining the separation power of high-performance anion exchange chromatography with the high sensitivity of pulsed amperometric detection. This innovative approach enables efficient and accurate qualitative and quantitative analysis of food ingredients and pharmaceutical impurities. Furthermore, its potential for quality control of food supplements and nutraceuticals has been demonstrated (64). The utilization of HPAE-PAD technology will contribute to the establishment of a regulatory system for food supplements and nutraceuticals, thereby reducing the risk of incorrect or ineffective food supplement interventions leading to the exacerbation of KOA. This will help alleviate pressure on the KOA healthcare sector.

## 5 Conclusion

The findings of this bibliometric study synthesized the closely interlinked and significant signaling pathways of the NF-κB signaling pathway in the pathogenesis of KOA with the aim of promoting further in-depth research in this field. In the study of signaling pathways in KOA, particular attention should be paid to the specific miRNAs and exosomes in BMSC, which will facilitate the development of a cure for KOA.

The bibliometric analysis revealed that studies of NF-κB signaling pathways in KOA have predominantly focused on the TLR4/NF-κB signaling pathway and exosomes. NF-κB plays a core regulatory role in KOA, with molecular evidence supporting its involvement in inflammation, cartilage degradation, and pain signaling. However, current research is limited by the lack of *in vivo* imaging techniques to visualize NF-κB activity in real-time. The development of such imaging modalities is crucial for understanding the dynamic changes in NF-κB activation during disease progression and for evaluating the efficacy of therapeutic interventions targeting this pathway.

Future research should prioritize the integration of multi-omics approaches, including single-cell and spatial transcriptomics, to analyze pathway heterogeneity. Single-cell transcriptomics can provide insights into the cellular diversity and specific cell types involved in NF-κB signaling, while spatial transcriptomics can reveal the spatial distribution of NF-κB activity within the joint tissue. This integrative approach will facilitate a deeper understanding of the pathogenesis of KOA and enable the identification of novel therapeutic targets and biomarkers.

While significant progress has been made in understanding the role of NF-κB in KOA, further research is needed to overcome current limitations and leverage advanced technologies to advance the field. This will ultimately lead to the development of more effective treatment strategies for patients suffering from KOA.

Furthermore, a summary of the most commonly used drugs, equipment, and research methods in the laboratory in the field of KOA can provide consultation for subsequent researchers and facilitate the advancement of experimental and application areas in KOA. As a result of the diversification of KOA treatments, food supplements, and nutraceuticals as alternatives to KOA drugs, these products are expected to become increasingly prevalent. Therefore, it is essential to reinforce supervision and management in order to prevent adverse events.

## 6 Limitations

The papers searched for in this study were obtained from the WoSCC and may have missed quality papers from other databases. In addition, the WoSCC is constantly being updated, and there may be a lag in the papers included in this study. However, omitting a limited number of papers does not substantially influence the status and trajectory of research in this field. Nevertheless, more authoritative databases and newer studies will be included to further improve the quality of research in more in-depth studies in the future.

## Data Availability

The original contributions presented in this study are included in this article/supplementary material, further inquiries can be directed to the corresponding author.

## Footnotes

1
http://bibliometric.com/
