# Correction: A bibliometric analysis of studies related to the nuclear factor kappa b signaling pathway in knee osteoarthritis between 2004 and 2023

**DOI:** 10.3389/fmed.2025.1658371

**Published:** 2025-07-17

**Authors:** Yafeng Mo, Jiahao Chu, Tiewu Chen, Wenbing Liu, Jing Tang

**Affiliations:** ^1^Department of Orthopedics, Zhejiang Rehabilitation Medical Center, Hangzhou, China; ^2^Department of Orthopedics, The Third Affiliated Hospital of Zhejiang Chinese Medical University, Hangzhou, China

**Keywords:** knee, osteoarthritis, NF-κB, signaling pathway, bibliometric analysis

In the published article, there was an error in the Funding statement. In the sentence “...., the Pillar Talent Project of the Excellent Talent Cultivation Program of Zhejiang Rehabilitation Medical Center (Zhejiang Rehabilitation Medical Center, 2024, No. 10).”, the text in parentheses “(Zhejiang Rehabilitation Medical Center, 2024, No. 10)” should be removed. The correct Funding statement appears below.

